# A community-based cluster randomised controlled trial in rural Bangladesh to evaluate the impact of the use of iron-folic acid supplements early in pregnancy on the risk of neonatal mortality: the Shonjibon trial

**DOI:** 10.1186/s12889-018-5713-1

**Published:** 2018-07-03

**Authors:** Tanvir M. Huda, Mohammad Masudur Rahman, Shahreen Raihana, Sajia Islam, Tazeen Tahsina, Ashraful Alam, Kingsley Agho, Sabrina Rasheed, Alison Hayes, Mohd Anisul Karim, Qazi Sadequr Rahman, Abu Bakkar Siddique, Md Moinuddin, Morseda Chowdhury, Lucky Ghose, Kaosar Afsana, Camille Raynes-Greenow, Shams El Arifeen, Michael J. Dibley

**Affiliations:** 1 School of Public Health, Faculty of Medicine and Health, The University of Sydney, Sydney, NSW Australia; 2 Maternal and Child Health Division, International Centre for Diarrhoeal Disease Research, Dhaka, Bangladesh; 3 Norman J Arnold School of Public Health University of South Carolina, Columbia, USA; 4 School of Science and Health, Western Sydney University, Sydney, Australia; 5 Health System and Population Studies Division, International Centre for Diarrhoeal Disease Research, Dhaka, Bangladesh; 6 Nuffield Department of Population Health, University of Oxford, Oxford, England; 7 Department of Statistical Science, University of Padova, Padova, Italy; 8 Health Nutrition and Population Programme, BRAC, Dhaka, Bangladesh

## Abstract

**Background:**

Iron-deficiency is the most common nutritional deficiency globally. Due to the high iron requirements for pregnancy, it is highly prevalent and severe in pregnant women. There is strong evidence that maternal iron deficiency anaemia increases the risk of adverse perinatal outcomes. However, most of the evidence is from observational epidemiological studies except for a very few randomised controlled trials. IFA supplements have also been found to reduce the preterm delivery rate and neonatal mortality attributable to prematurity and birth asphyxia. These results combined indicate that IFA supplements in populations of iron-deficient pregnant women could lead to a decrease in the number of neonatal deaths mediated by reduced rates of preterm delivery. In this paper, we describe the protocol of a community-based cluster randomised controlled trial that aims to evaluate the impact of maternal antenatal IFA supplements on perinatal outcomes.

**Methods/design:**

The effect of the early use of iron-folic acid supplements on neonatal mortality will be examined using a community based, cluster randomised controlled trial in five districts with 30,000 live births. In intervention clusters trained BRAC village volunteers will identify pregnant women & provide iron-folic acid supplements. Groundwater iron levels will be measured in all study households using a validated test kit. The analysis will follow the intention to treat principle. We will compare neonatal mortality rates & their 95% confidence intervals adjusted for clustering between treatment groups in each groundwater iron-level group. Cox proportional hazards mixed models will be used for mortality outcomes & will include groundwater iron level as an interaction term in the mortality model.

**Discussion:**

This paper aims to describe the study protocol of a community based randomised controlled trial evaluating the impact of the use of iron-folic acid supplements early in pregnancy on the risk of neonatal mortality. This study is critical because it will determine if antenatal IFA supplements commenced in the first trimester of pregnancy, rather than later, will significantly reduce neonatal deaths in the first month of life, and if this approach is cost-effective.

**Trial registration:**

This trial has been registered with the Australian New Zealand Clinical Trials Registry (ANZCTR) on 31 May 2012. The registration ID is ACTRN12612000588897.

## Background

Globally, 2.6 million children died in the neonatal period, the first 28 days of life in 2016 [1]. Over the last two decades, there has been a substantial global reduction in under-5 child mortality rates. Compared to that, the reductions in neonatal deaths have been slower. Neonatal deaths accounted for 46% of all under-five deaths in 2016 compared with 37·4% in 1990 [1, 2]. Effective interventions directed specifically at preventing neonatal deaths are required to improve overall child survival.

A systematic review in 2005 found that up to 70% of neonatal deaths, worldwide, could be averted by implementing sixteen evidence-based interventions at near universal coverage (99%) that incorporated preconception, antenatal, intrapartum and postnatal interventions [3]. However, this review did not include antenatal iron and folic acid (IFA) supplementation for pregnant women. At that time no trials had reported the effects of IFA on neonatal mortality, although these supplements were known to benefit both maternal and infant health [3].

Iron-deficiency is the most common nutritional deficiency globally. Due to the high iron requirements for pregnancy, it is highly prevalent and severe in pregnant women [4]. In Bangladesh, 42% of all women of reproductive age and nearly 50% of pregnant women had anaemia in 2011 [5]. However, the prevalence of iron deficiency was lower. The first national micronutrients status survey, published in early 2013, reported that iron deficiency among non-pregnant non-lactating women was 7.1%. The ferritin level was significantly higher in areas where groundwater iron concentration was high. The survey findings also suggest a positive association between natural iron content in groundwater and iron status of women [6].

There is strong evidence that maternal iron deficiency anaemia increases the risk of adverse perinatal outcomes. However, most of the evidence is from observational epidemiological studies except for a very few randomised controlled trials. A community-based cluster randomised trial from China reported a 54% reduction of early neonatal mortality in newborns of women who received IFA supplements compared to those receiving folic acid alone (HR 0.46, 95%CI: 0.21–0.98) [7]. Secondary analyses of this trial found a 71% reduction in neonatal mortality in women who started IFA supplements in the first trimester of pregnancy (HR 0.29, 95%CI: 0.09–0.88) [8]. A meta-analysis of prospective observational studies, reported reductions in perinatal mortality (stillbirth and early neonatal mortality) with each 1 g/dl increase in the population mean haemoglobin (RR 0.72, 95%CI: 0.65–0.81) [9]. A pooled analysis of data from Indonesian Demographic and Health Surveys, which included 40,576 singleton live-born infants, found mothers using any IFA supplements during pregnancy significantly reduced the risk of early neonatal death of their newborns after adjustment for potential confounders [10]. Similarly, a pooled analysis from Pakistan on 8512 live births reported maternal antenatal IFA supplements significantly reduced the adjusted risk of death on day 0 by 33% and during the neonatal period by 29%. Initiating IFA supplements in the first four months of pregnancy, significantly reduced the adjusted risk of neonatal mortality by 35% [11].

IFA supplements have also been found to reduce the preterm delivery rate and neonatal mortality attributable to prematurity and birth asphyxia [7]. Other studies from low-income countries have reported that preterm delivery is one of the main contributors to neonatal deaths [12, 13]. These results combined indicate that IFA supplements in populations of iron-deficient pregnant women could lead to a decrease in the number of neonatal deaths mediated by reduced rates of preterm delivery. We thus propose a large-scale prospective trial to measure the impact of the early introduction of antenatal iron/folic acid supplementation in pregnancy on neonatal outcomes in rural Bangladesh. We will employ a community-based, cluster randomized controlled trial design, in which pregnant women will be randomly allocated to the enhanced iron/folic acid distribution program, or to the current usual programs. We have chosen the current usual programs as our comparator. We didn’t consider placebo because WHO and Government of Bangladesh have a recommendation that pregnant women should receive 60 mg of iron supplementation. But the implementation of this recommendation is inadequate. Surveys indicate that only 52% of rural women reported any use of iron/folic acid in their last pregnancy but this dropped to 39% for the poorest households, and to 34% for women with no education (3). Also, the median gestation when women start antenatal care (and would have access to iron/folic acid supplements) is 5.2 months (3) .

### Objectives and hypothesis

The trial objectives are to evaluate the effect of early availability and promotion of IFA supplements on neonatal mortality, low birthweight and preterm delivery. The primary hypothesis is that in a community-based, cluster randomized controlled trial of women from rural Bangladesh, daily supplementation with 60 mg elemental iron and 400 μg folic acid starting in the first trimester of pregnancy, and sustained for at least 180 days, will reduce neonatal mortality by 25% from 33/1000 to 24.8/1000 live births compared to usual iron-folic acid supplementation programs.

The secondary hypotheses are that the early availability and promotion of IFA supplements in intervention clusters will reduce preterm delivery by 30% (15% in control to 10.5% in intervention), and low birth weight by 30% (15% in control to 10.5% in intervention). We further hypothesised that household wealth and maternal education would modify the neonatal mortality responses with a greater reduction for women from the poorest households, and women with no education. We also hypothesised that the early start to IFA supplements would not increase iron-related side effects, e.g. nausea & vomiting in intervention clusters and will be cost-effective in reducing neonatal mortality in intervention clusters.

## Methods

### Study design

This study is a cluster randomised controlled trial, with 2-arm parallel groups, superiority design and 1:1 allocation ratio. The cohort of the mother-child dyads will be followed up from recruitment until 42 days after birth.

### Study setting

**Fig. 1:**
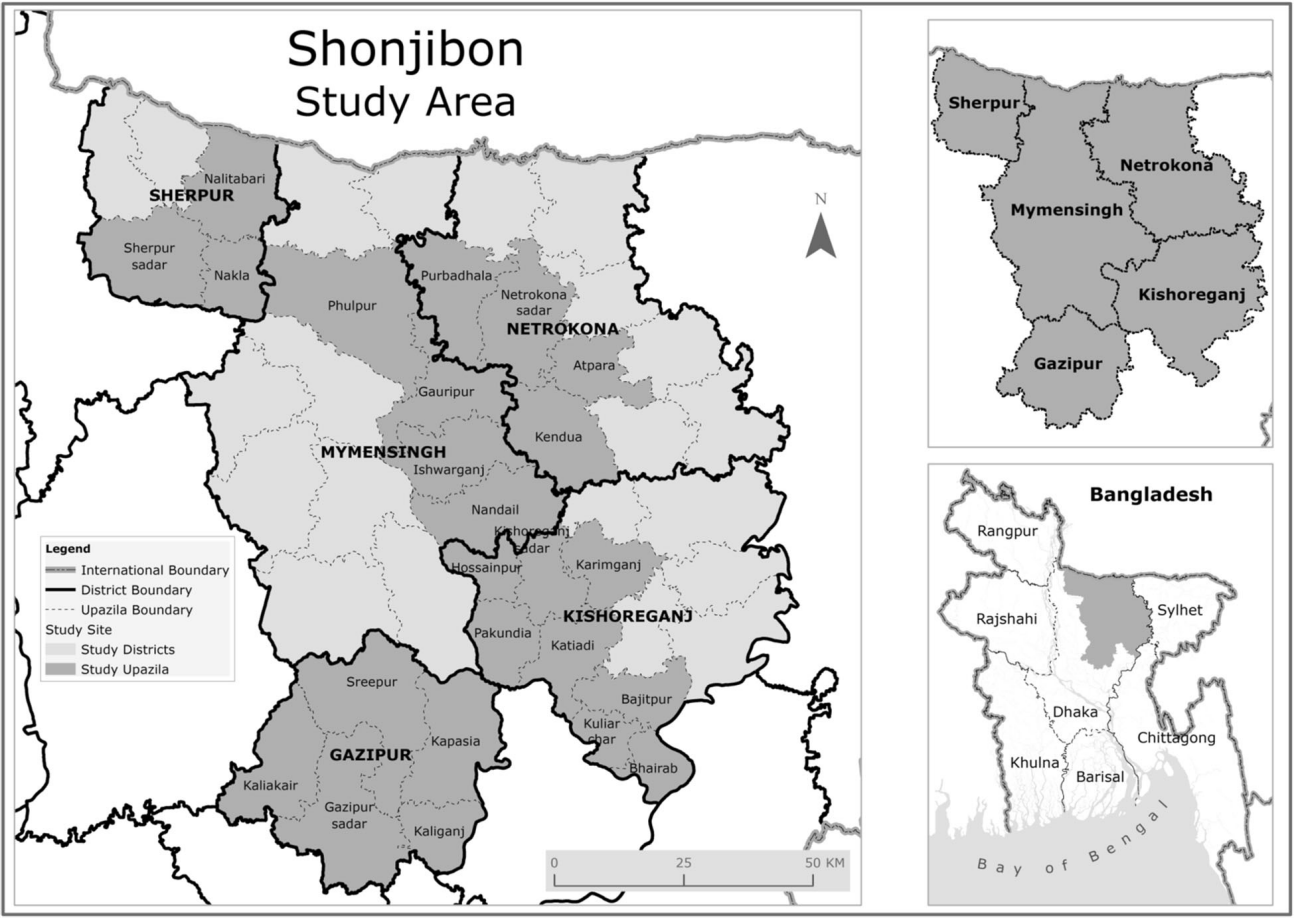
Map of study area, Shonjibon Trial (created by the authors)

We will conduct the Shonjibon trial in five districts in Dhaka Division, Bangladesh. These districts are Netrokona, Kishoreganj, Mymensingh, Sherpur, and Gazipur, with a total number of 47 Upazila (subdistricts) and a combined population of 20 million. Our study implementing partner BRAC, a non-government organisation co-ordinates a community-based maternal, neonatal and child health program in the area which includes a prospective pregnancy surveillance and home-based antenatal care program. The Shonjibon study area will consist of 24 Upazilas from these five districts (Fig. 1). We will exclude five Upazilas from Kishoreganj, six Upazilas from Netrokona and two Upazilas from Sherpur because of insufficient BRAC Program Officer (PO) coverage. We will also exclude three Upazilas from Gazipur because of the high number of garment industries, which would have high numbers of working women and would be difficult to follow-up and eight Upazilas from Mymensingh because of the high prevalence of malaria and kalazar.

### Cluster selection

We will define a cluster as a BRAC PO area, which will consist of a pair of BRAC village field workers (called Shasthya Kormi or SK) and their 20 BRAC village volunteers (referred to as Shasthya Shebika or SS) who are supervised by a single BRAC PO. A BRAC PO area approximately comprises of 40 to 50 villages with an average population of 13,000 people. We will have a total number of 112 BRAC PO for randomisation. We will randomly select 100 clusters with an equal number of clusters in each arm. The research team took written approval for the study from Directorate General of Health Service (DGHS) under Ministry of Health & Family Welfare before allocating the clusters to a treatment group or the trial interventions begin.

### Eligibility criteria for inclusion of cluster

We will exclude any potential BRAC PO area (cluster) if there are any other maternal and or neonatal interventions either by the government or non-government organisations in the area. We will identify any such program by contacting local officials in the sub-districts in the Dhaka Division. We will exclude areas where access is extremely difficult, for example in flood-prone areas. All women aged between 15 to 49 years, who are pregnant and their gestational age is ≤90 days, and are permanent residents of the Shonjibon study area will be eligible for the study.

#### Formative research

Before the intervention started, we conducted a formative study using qualitative methods. We collected the data from July to October 2012, in two villages, one in Mymensingh and one in Netrokona – two of the five districts included in the Shonjibon Trial. The field team consisted of three women and two men trained in anthropology or social sciences, who had experience in collecting qualitative data. Four qualitative methods – in-depth interviews (35 pregnant women, 20 older women and 11 husbands), key informant interviews (20 with CHWs, program managers, informal care providers, gynaecologists and traditional birth attendants), focus group discussions (2 with Shasthya Shebika and 1 with female college students) and observations of the lowest level of health facility (community clinics) (2) were conducted. We explore women’s and family members’ perceptions of: cultural norms during pregnancy; birth weight; IFA supplementation; and experience using IFA supplements during pregnancy. We conducted the key informant interviews to understand the community norms around IFA supplementation, and challenges and successes of the existing programme. We used FGDs to understand both perceptions regarding the need for IFA tablets during pregnancy and the state of IFA supplementation programmes. In two community clinics, we conducted an hour-long observation to understand the implementation of the national IFA programme.

#### Intervention

From the formative study, we identified culturally appropriate and feasible ways of implementing the intervention [14]. The findings of the formative study suggested that introduction of IFA supplements early in pregnancy is feasible with support from BRAC or government community health workers. The formative study also suggested to include culturally sensitive reasons for the use of IFA supplements during pregnancy, improvement of the community health-worker training modules and to standardise messages across all health workers. We conducted a pilot study in four BRAC village fieldworker areas, two with intervention and two comparison areas, to test the feasibility of our intervention.

### Early identification of pregnancy

In the intervention clusters, trained SS will identify pregnant women through systematic door-to-door visits of the households in her catchment area and follow-up with all women of reproductive age about their recent history of missed menstrual periods. We will financially incentivise the SS for early identification of pregnant women.

### Regular supply of IFA supplements and counselling

Upon receiving the consent icddr,b study staff (Field Implementers or FI) will hand over the first two months of supply of iron-folic acid capsules, each containing 60 mg of elemental iron and 400μg of folic acid. She will also counsel the mother about the benefits of IFA supplements in pregnancy. The SS will then make regular fortnightly visits to the woman until delivery to ensure resupply of supplements, and provide counselling in support of early uptake, continued use, and compliance with the supplementation regime until delivery. This intervention is consistent with the Bangladesh Ministry of Health guidelines.

### Improve adherence and monitor compliance

To improve adherence, a local pharmaceutical company (Eskayef Bangladesh Limited) will produce IFA supplements in an attractive capsule shell and special blister package. Each strip will consist of 15 capsules. At the time of enrolment, we will assign each mother a box consisting of 8 months of supply of iron-folic acid supplements. We will attach the details of the pregnant women to the box during registration in the trial. At the time of resupply, the SS will collect the empty strips from the previous round. If she finds any unused capsules, she will inquire about the missing doses and further counsel the women about the importance of regular consumption of IFA supplements. We will provide a specially designed medicine box to each participant to preserve the medicine. It will also serve as a reminder for regular consumption and safely store the medicines away from child access. In addition to this, a special call centre will be set up centrally where trained nutrition counsellors will systematically make bi-weekly calls to monitor compliance with the supplementation regime.

### Concomitant treatment

The research team will advise all Shonjibon study participants in the intervention arm not to consume any additional iron or folic acid supplements from other sources during her pregnancy.

### Management and training

Overall around 3920 SS and 100 FI will deliver the intervention, under the supervision of central field supervisory team. There will be one FI for each cluster. The central team will comprise of three research fellows, one medical officer and one research investigator and one BRAC national manager. The field supervision team will comprise of five Field Research Officers and five District Managers. We will train the BRAC village workers and volunteers in sub-district health centres to detect and monitor pregnancies, to maintain pregnancy records, to manage supply and distribution of supplements, communication and counselling skills for behaviour change related to use of iron-folic acid supplements. The central team will train the field team and monitor the process of delivery of the intervention. The BRAC PO will provide technical support to the SK and SS, and will help them resolve any problems through regular meetings in the field.

#### Control

We used usual iron supplementation program as the comparator. We will identify and confirm pregnant women in the control clusters using methods similar to those described for the intervention arm. Women will not receive any additional information or counselling other than usual standard care provided by the existing BRAC structure. Both intervention and control treatment arms will receive the usual antenatal and postnatal care services by BRAC.

#### Trial outcomes

The primary trial outcome will be the difference in the neonatal mortality rate between the intervention and control areas. The secondary trial outcomes will include: (1) percentage of women using IFA supplements in the first trimester of pregnancy; (2) percentage of live births with low birth weight and with preterm delivery; (3) percentage of neonatal deaths attributable to preterm delivery, and to birth asphyxia; (4) mean marginal additional expenditure associated with early IFA supplements, and the mean cost per neonatal death prevented; (5) the percentage of pregnant women reporting side effects including nausea and vomiting.

### Outcome measurements

#### Neonatal deaths

We will investigate all neonatal deaths reported by the SS to the Field Implementers or by maternal recall at the final follow-up visit at 42 days postpartum, using standard questionnaires collected by trained staff. We will use this data to calculate estimates of neonatal mortality in both arms from baseline to the end of the intervention period.

#### Verbal autopsy

Trained Field Research Officers will interview caretakers of infants who die in the neonatal period using the World Health Organization (WHO) Standard Verbal Autopsy Questionnaire [15] within one month of the death. Investigators will extract and code the symptoms from both the closed-ended questions and open narratives. We will use physician-certified verbal autopsy method to identify cases of death related to preterm delivery and birth asphyxia using the hierarchical assignment of causes of death. At least two physicians will independently review the data, and they will base their decision on an agreement. Review by a third physician will resolve any discrepancy between the two diagnoses.

#### Birth weight

We will measure birth weight within 24 h of delivery using standard methods with a portable electronic scale. The SS will notify the Field Implementers of any births in their area, and both will visit the mother and her infant within 24 h, although measurements will be accepted up to 72 h after birth.

#### Duration of gestation

We will measure the duration of gestation will be measured using the date of last menstrual period with the pregnancy confirmed by urine test in the field.

#### Other measurements

Social, economic and demographic characteristics will be collected in a questionnaire collected at enrolment and will use standard demographic and health survey methods of an inventory of household assets to construct a wealth index, [16] as well as the socio-demographic characteristics of the mother.

#### Cost-effectiveness analysis

The economic evaluation will take a societal perspective. We will base the data for the cost-effectiveness analysis on the costs and outcomes observed in the trial. The costs of the intervention will include cost and distribution of the supplements, training of SS workers, organisational costs associated with the administration of the programme and the cost of incentives. We will ask the participants to record use of health services at each follow-up interview, which will enable assessment of potential cost offsets particularly associated with differences in rates of birth complications and neonatal morbidity. We will estimate incremental cost-effectiveness ratios as incremental cost per neonate death avoided; per low birth weight infant avoided and per life-year saved. Years of life saved will be based on any difference in survival of mothers and infants observed during the trial and estimated using current life expectancy in Bangladesh. Standard methods for estimating uncertainty and a cost-effectiveness acceptability curve will be generated to provide information on the probability that the intervention is cost-effective, at different willingness to pay thresholds.

#### Groundwater iron

We will measure the level of groundwater iron in all the households of our study using a validated test kit tool [±0.1 mg/L, FerroVer method, HACH Iron Test kit, Model IR-18B].

### Sampling frame and sample size

To detect a 25% reduction in neonatal mortality (33 neonatal deaths/1000 live births in control to 24.8 neonatal deaths/1000 live births in the intervention group), with 90% power, 5% two-sided alpha, 1:1 ratio of treatment allocation and intra-cluster correlation coefficient (ICC) of 0.0245, we require 100 clusters (each covering an average of 4000 households), or 50 clusters per treatment group, with a total of 30,000 live births over one year. This sample would allow detection of a 22% relative difference between treatment groups with 80% power. To detect a 30% reduction in preterm delivery from 15% in control compared to 10.5% in the intervention arm, assuming 80% power, 5% level of significance, intracluster correlation of 0.012 for small birth size (Bangladesh DHS data), would require a sample size of 1300/arm or 40 pregnant women from 64 clusters. A smaller sample size of 570 women/arm or 40 pregnant women from 28 clusters is needed to detect a 30% reduction in LBW with the same assumptions. Our cluster is one BRAC PO area that consists of ~ 13,000 people. Assuming a crude birth rate of 23 per 1000 people, 10% loss of pregnancies before birth and 15% loss to follow up each cluster will yield 300 births over 15 months, and this will be sufficient to reach the desired sample size.

### Recruitment and consent of women

In the intervention clusters, SSs will make systematic door-to-door visits to identify new pregnancies through menstrual cycle tracking of all women of reproductive age. Once the woman reports a missed period, the SS will immediately notify the Field Implementers by mobile phone. The Field Implementers will then visit the pregnant mother along with the SS within the next 48 h to conduct a pregnancy test and enquire about the last menstrual period. If the pregnancy test is positive and the pregnancy is within the first trimester, the Field Implementers will register the mother and seek written consent. We will obtain written, witnessed informed consent from all participants.

### Assignment of treatments

An investigator remote from the field area will assign the interventions to eligible clusters using a fixed randomisation scheme with a uniform allocation ratio of treatments, stratified by subdistrict or Upazila. The random allocation sequence will be generated using Stata 12 software (StataCorp. 2011. Stata Statistical Software: Release 12. College Station, TX: StataCorp LP). The nature of the intervention precludes the masking of the treatments.

### Evaluation

#### Data collection team

A separate team of trained data collectors not involved in the implementation of the intervention will collect data for evaluation. The evaluation team will consist of 135 data collectors, five field supervisors and five field research officers. Each data collector will be responsible for one cluster. We will train the evaluation team over a four-week period, and standardize measurement methods and retrain as needed every six months.

#### Data collection schedule

**Fig. 2:**
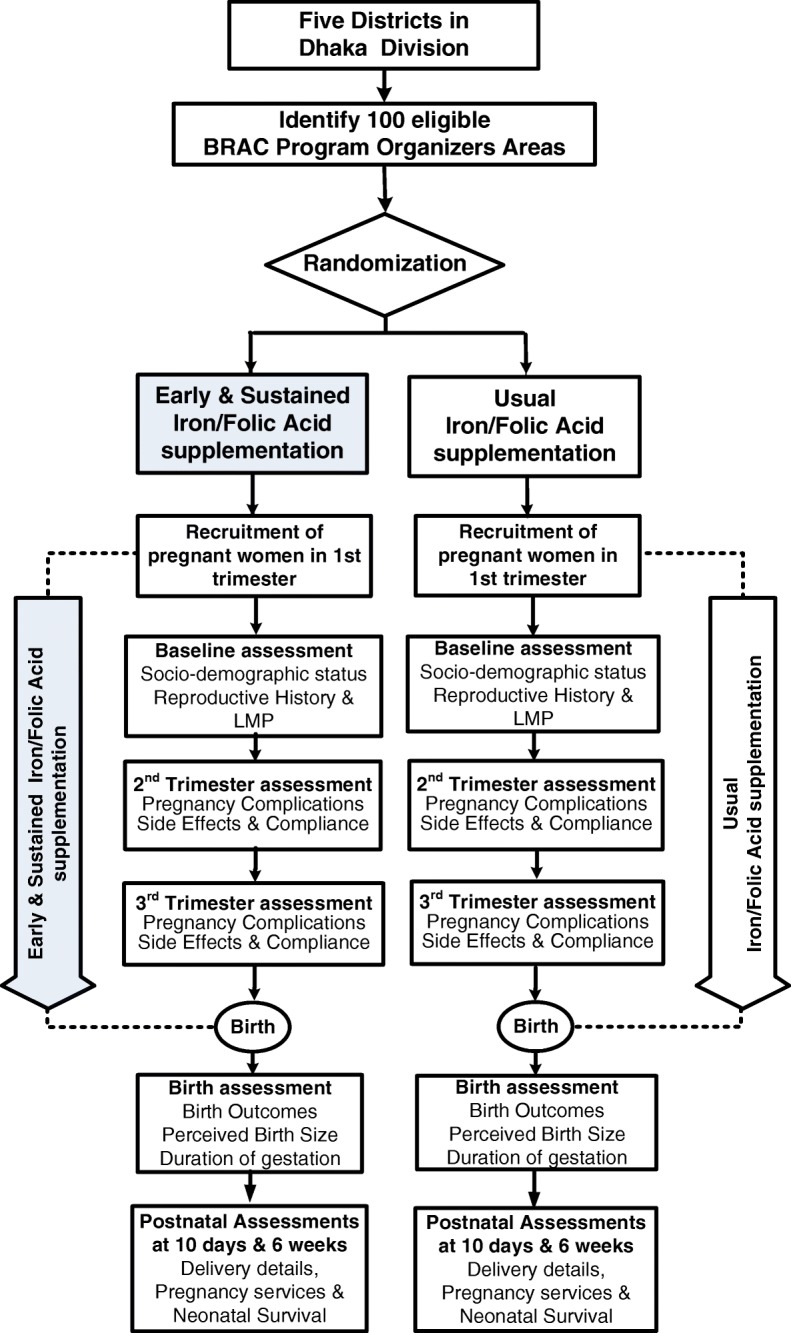
Trial flow chart and intervention schedule

The data collectors will visit each woman a total number of six times from the time of enrolment till 42 days after delivery. These visits will be made between 12 and 14 weeks, 20–24 weeks, 28–32 weeks, within 24 h of delivery, 7–10 days after delivery, 38–42 days after delivery (Fig. 2).

#### Types of information collected

Over the pregnancy and neonatal period, data collectors will collect information regarding household characteristics, reproductive history, pregnancy information, medication history, details about consumption of IFA supplements, current health status, any side effects from the supplements, antenatal care, birth outcome, birth weight, immediate newborn care and neonatal deaths. We will also collect information on exposure to household air pollution and out of pocket expenses for any antenatal and postnatal care seeking (Table 1).

**Table 1:**
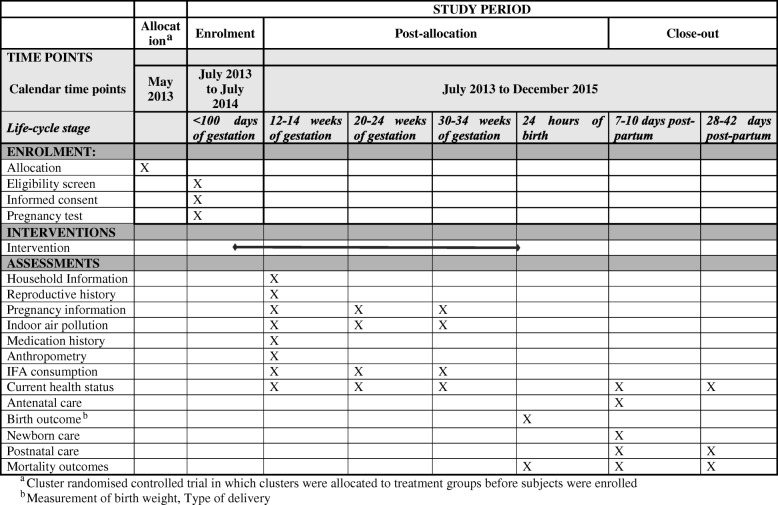
Schedule of enrolment, interventions, and assessments

#### Electronic data collection tool

Data will be captured using an android application developed using Java. Each data collector will use Samsung Galaxy 7 in. tablet with Internet enabled SIM card for data collection and transmission of data from the community to central database server. The application will navigate the data collectors through a step-by-step process while interviewing the pregnant women to collect all required information to be collected at each specific visit. Some validation rules will be set in the application to prevent errors during data collection. Rules will include logical and range checks, uniqueness check and skipping rules as appropriate.

Initially, the Field Implementers will enter all the required registration information of the woman into the system. As soon as the data is uploaded to the server, information will be passed to the separate evaluation team for first follow up visit. Each data collector will need to log into the system using a pre-assigned unique user ID and password. During the first visit, the data collector will download the details of the newly registered pregnant women for her assigned cluster from the central server. This information, filtered according to the geographical area, comes from the registration data collected by the Field Implementers. During the subsequent visits, the app will provide each data collector with a list of women, for whom a follow-up visit is scheduled within seven days. We will use different colour coding to highlight visits that are pending or imminent. To verify physical presence of the data collectors at the households we will record a Geographic Information System (GIS) data point. The system will also generate daily reports of the completed interviews and enrolment and make them available on the online monitoring interface (in real time). At the end of each interview, the data collector will store the data on the local device and then upload the data to the central server after the end of a day’s work. We will maintain a backup copy of the database on a different server.

### Statistical analysis

Data analysis will be by intention to treat. We will conduct analyses at the infant level adjusted for the cluster randomisation. The primary analyses will compare the neonatal mortality rates and their 95% confidence intervals adjusted for clustering and groundwater iron level in each group. Secondary analyses will examine each outcome variable using separate mixed models. We will use Cox proportional hazards mixed models for mortality outcomes and include groundwater iron level as an interaction term in the mortality model. We will use population average Poisson model for other outcomes including preterm, small for gestation and low birth weights. Models will include treatment group as a fixed effect, community-cluster as a random effect to account for the cluster effect. The models will evaluate the impact of the interventions over time by testing for an interaction between time and intervention group. We will conduct analyses to identify the subgroups (based on household wealth, sex of infant and maternal education) that modify the response to the intervention. We will check the model assumptions and make appropriate adjustments to the analysis where necessary. Descriptive analysis will be performed to present the cause of death. We will compare the causes of death by sex of newborn, the time-period of death, and treatment arms (intervention Vs control). χ^2^ test or Fisher’s Exact test after adjusting for cluster effect will be used when appropriate. The economic analysis will estimate the incremental cost per life saved per year of the intervention over standard care. We will use sensitivity analyses of the findings to assess uncertainty in key parameters. Stata V.14 (StataCorp. 2015. Stata Statistical Software: Release 14. College Station, TX: Stata Corp LP.) will be used for all analyses.

### Data monitoring

#### Electronic data monitoring tool

We will develop an online monitoring system, which will provide us real-time information on enrolment status, adherence of the data collectors with the evaluation schedule and quality of data for key variables. This system will allow the field supervisors to monitor the performance of individual data collectors. All reports will be auto-generated from the system and will be available anytime for the investigators’ assessment.

#### Data safety and monitoring board

The research team will establish an independent data safety and monitoring board (DSMB) consisting of an independent paediatrician, a clinical trial specialist and a biostatistician. This DSMB is responsible for assessing the interim data, data quality, data completeness, adequacy of compliance with goals for recruitment and retention, and factors that might affect the study outcome or compromise the confidentiality of the trial data. Any unintended effects of trial intervention will also be notified to data safety and monitoring board.

#### Advisory committee

To facilitate the overall implementation process and to engage policymakers and other key decision makers, we will establish a national project advisory committee consisting of Ministry of Health and Family Welfare staff, staff, representatives from UNICEF, WHO, the Obstetric and Gynaecological, the Perinatal, and the Nutrition Societies of Bangladesh. This committee will assist in translating the Shonjibon study findings into policy. In addition, we will establish five district advisory committees consisting of local health officers, local government officials, local community groups, other relevant NGOs and project staff to help guide the research team.

### Access to data

All collected data will be accessible by the study investigators. All the investigators will have the right to analyse and publish data. We will only share datasets with all personally identifiable information removed, and every reasonable effort to keep the identification of study subjects in the strictest confidence.

### Dissemination plan

The research team will share the lessons learned from the study widely throughout Bangladesh and among global audiences. The authors will organise dissemination seminars to share the findings with all relevant stakeholders. We will present the intermediate and immediate results, which support the hypotheses generated from the trial, at national and international conferences and publish them in conference proceedings. We will publish the analysis of the outcomes in the form of internal documents, working papers, and in international peer-reviewed journals. Authorship eligibility will be based on recommendation from International committee for medical journals editors (ICMJE).

## Discussion

Iron deficiency is associated with increased risk of maternal death, preterm delivery, impaired foetal growth and development, low birth weight, increased neonatal mortality and impaired infant development [17]. In resource-poor countries, rates of maternal anaemia remain high. Although the importance of IFA supplements to prevent maternal anaemia and mortality is widely accepted, most antenatal IFA supplements distribution programs are of low priority. Evaluations of these programs using maternal anaemia as the outcome report little or no impact because of the multiple causes of maternal anaemia, low compliance, and inadequate distribution programs.

Recent surveys in Bangladesh indicate that only 52% of rural women reported any use of IFA supplements in their last pregnancy, but this falls to 39% for the poorest households, and to 34% for women with no education [5]. In the Projahnmo trial conducted in rural Bangladesh, only 40% of women in the baseline surveys reported any use of IFA supplements in pregnancy [18]. Access to IFA supplements is through antenatal care, however, in rural Bangladesh, the median gestation that women commence antenatal care is 5.2 months, and 44% of women report no use of antenatal care [5]. The Projahnmo trial demonstrated improved IFA supplements as 84% of pregnant women in the home-care treatment arm reported IFA supplements use in pregnancy [18]. Although they did not start early, these figures indicate there is a substantial opportunity to improve IFA supplements distribution in rural Bangladesh.

There is limited evidence about the optimal timing of IFA supplementation in pregnancy. Promising evidence came from a trial in China investigating the effects of IFA supplements in pregnancy on neonatal deaths [19]. However, the neonatal mortality analyses were post-hoc analyses, and the sample size was small resulting in poor precision in the effect estimates, and reduced power to explore all the factors modifying the effects on neonatal mortality. Thus, the impact of early IFA supplements on neonatal mortality needs confirmation in a larger scale trial.

This project will determine if antenatal IFA supplements commenced in the first trimester of pregnancy, rather than later, will significantly reduce neonatal deaths in the first month of life, and if this approach is cost-effective. It will also provide valuable evidence about the role of groundwater iron level in modifying the responses of antenatal IFA supplements on neonatal mortality since there is already some evidence which suggests a positive, dose-response association between natural iron in groundwater and the iron status of women [6].

## Abbreviation

ERC: Ethical Review Committee
HREC: Human Research Ethics Committee
IFA: Iron and folic acid
NHMRC: National Health and Medical Research Council
